# Alterations in Red Blood Cells and Plasma Properties after Acute Single Bout of Exercise

**DOI:** 10.1155/2013/168376

**Published:** 2013-12-18

**Authors:** Krzysztof Gwozdzinski, Anna Pieniazek, Joanna Brzeszczynska, Sabina Tabaczar, Anna Jegier

**Affiliations:** ^1^Department of Molecular Biophysics, University of Lodz, Pomorska 141/143, 90-236 Lodz, Poland; ^2^Department of Thermobiology, University of Lodz, 90-236 Lodz, Poland; ^3^Department of Sport Medicine, Medical University of Lodz, 90-647 Lodz, Poland

## Abstract

The aim of this study was to investigate alterations in haemoglobin conformation and parameters related to oxidative stress in whole erythrocytes, membranes, and plasma after a single bout of exercise in a group of young untrained men. Venous blood samples from eleven healthy young untrained males (age = 22 ± 2 years, BMI = 23 ± 2.5 kg/m^2^) were taken from the antecubital vein before an incremental cycling exercise test, immediately after exercise, and 1 hour after exercise. Individual heart rate response to this exercise was 195 ± 12 beats/min and the maximum wattage was 292 ± 27 W. Immediately after exercise, significant increase in standard parameters (haemoglobin, haematocrit, lactate levels, and plasma volume) of blood was observed as well as plasma antioxidant capacity one hour after exercise. Reversible conformational changes in haemoglobin, measured using a maleimide spin label, were found immediately following exercise. The concentration of ascorbic acid inside erythrocytes significantly decreased after exercise. A significant decline in membrane thiols was observed one hour after exercise, but simultaneously an increase in plasma thiols immediately after and 1 h after exercise was also observed. This study shows that a single bout of exercise can lead to mobilization of defensive antioxidant systems in blood against oxidative stress in young untrained men.

## 1. Introduction

A substantial amount of evidence in the existing literature indicates that exercise is associated with increased generation of reactive oxygen species (ROS), including nitrogen and chlorine species [1, 2]. This increase appears to be due to disruption of the oxidant-antioxidant homeostasis and results in induction of oxidative stress in the human body. In humans, exercise has been reported to increase skeletal muscle superoxide dismutase (SOD) activity and the activity of various protective enzymes in the blood. Reactive oxygen species generated during exercise can cause oxidative stress and damage to structural and functional integrity at the cellular level. Moreover, there is no doubt that moderate exercise has numerous health benefits and that regular physical activity is an important factor in the prevention and treatment of cardiovascular diseases. It improves physical and psychological well-being as well as delays the ageing process [3, 4].

Oxygen consumption during exercise increases ten- to fifteenfold, but oxygen supply to active tissue may rise by a factor of one hundred [5]. ROS production in response to strenuous exercise can proceed through different ways: the mitochondrial respiratory chain or NAD(P)H oxidase, xanthine oxidase, and autooxidation of catecholamines [6]. ROS can also be released by macrophages and neutrophils in a respiratory burst or during destruction of iron-containing proteins [7, 8]. Moreover, increased production of ROS has been observed in both strenuous prolonged exercise or brief maximal exercise [9, 10]. In the case of intensive exercise, a high level of oxygen consumption, mechanical (shear) stress, disruption of iron-containing proteins, and an increase in prostanoid and catecholamine levels have been detected [11].

In our previous work, we examined the effect of exercise-induced oxidative modifications on the physicobiochemical properties of erythrocyte membrane in young untrained males [12]. However, there is little evidence concerning the influence of physical activity on the disintegration of internal erythrocyte components. In addition, it is not known whether alteration in haemoglobin conformation occurs during exercise. However, it has been reported that 3% of total haemoglobin (5 mmol/L) is converted daily to methaemoglobin, releasing superoxide anion radicals [13]. As a precursor of other reactive oxygen species, superoxide is continuously produced in erythrocytes due to a high oxygen tension in arterial blood and haeme iron content, which acts as a free radical catalyst [14]. Under these conditions, ROS can damage erythrocyte components and, after leaving the cell, these have the potential also to damage other plasma components in circulation [15]. This paper is focused primarily on the modulation of the conformational properties of internal cellular components (mainly haemoglobin) following a single bout of exercise in young, untrained males. It seems that a higher consumption of oxygen and free radical generation can lead to changes in haemoglobin conformation during exercise. As an indicator of the internal antioxidant system, ascorbic acid and glutathione concentrations inside erythrocytes were measured, as well as parameters related to oxidative stress in the cellular membrane. Changes in the erythrocyte membrane and haemoglobin conformation were examined against the background of plasma parameters.

## 2. Materials and Methods

### 2.1. Chemicals

4-Maleimido-2,2,6,6,-tetramethylpiperidine-1-oxyl (MSL), 4-iodoacetamide-2,2,6,6,-tetramethylpiperidine-1-oxyl (ISL), and 4-amino-2,2,6,6-tetramethylpiperidine-1-oxyl (Tempamine) were obtained from Sigma Chemical Co. (St. Louis, MO). All other chemicals were analytical grade products from POCh (Gliwice, Poland).

### 2.2. Subjects and Protocol

Eleven healthy males (mean age, 22 ± 2 years; mean height, 181 ± 7 cm; mean mass, 83 ± 8.5 kg; mean body mass index, 23 ± 2.5 kg/m^2^) volunteered to participate in this study. Subjects recruited were clinically healthy according to a medical doctor's examination that had the following exclusion criteria: resting blood pressure higher than 140/90, resting heart rate higher than 90 beats/min, smoking, or using antioxidant supplements and medicaments. All subjects were untrained (i.e., not performing any regular physical activity). Ethical approval was obtained from the Medical University of Lodz. All subjects signed an informed consent form prior to participation.

### 2.3. Experimental Procedures

Subjects refrained from performing strenuous exercise and drinking alcohol for 24 hours prior to testing and came to the lab in the morning after an overnight fast (no breakfast, tea or coffee).

Exercise was performed using a friction-braked cycle ergometer (Monark, Sweden). Subjects started pedaling at 60 Watts and 60 rpm (revolutions per minute) for 1 min, after which the workload was increased by 30 Watts every minute until volitional exhaustion. Subjects maintained a pedal frequency of 60 rpm throughout the test and they were verbally encouraged to cycle until exhaustion. The heart rate at peak of exercise was higher than 90% of the predicted maximal heart rate according to the subjects' age. Individual response to this exercise was HRmax 195 ± 12 beats/min, and maximum wattage (Wmax) was 292 ± 27 W; maximum wattage per kilogram was  3.43 ± 0.57 (W/kg). During the exercise test, heart rate was measured using ECG (system Case 16 DRG comp.). Total work performed during exercise amounted to 92782 ± 16465 J. Average time of exercise duration was 8.73 ± 0.9 min. The protocol used was the Bruce treadmill test protocol, as this is the method for estimating VO_2_ max⁡ in athletes. The VO_2_ max⁡ range 25.89−32.62 (mL/kg/min) was calculated according to the following formula (1):

(1) $$\begin{array}{c} \text{V}{\text{O}}_{2}\;\max=14.8-\left(1.379\cdot T\right)+\left(0.451\cdot T^{2}\right)-\left(0.012\cdot T^{3}\right) \end{array}$$

*T* is total time on cycle ergometer measured as a fraction of a minute.

Following exercise, subjects remained seated at rest for 1 hour, and were allowed to drink only water. This specific exercise protocol was used as a model of an acute stressor inducing oxidative stress.

Venous blood samples were taken from an antecubital vein before the exercise test, directly after exercise, and one hour after exercise. The plasma lactate concentration, haemoglobin (Hb) concentration, and haematocrit (HTC) were determined in the collected blood using autoanalyser. Hb and HTC were used to calculate the ΔPV value according to the Dill and Costill formula [16]:

(2) $$\begin{array}{c} \Delta \text{PV}=\left[\left(\frac{\text{H}{\text{b}}_{1}}{\text{H}{\text{b}}_{2}}\right)\cdot \left(\frac{100-\text{HT}{\text{C}}_{2}}{100-\text{HT}{\text{C}}_{1}}\right)-1\right]\cdot 100\%, \end{array}$$

Where  Hb_1_, HTC_1_ are preexercise values and Hb_2_, HTC_2_ are postexercise or 1 h recovery values.

For other experiments the blood was centrifuged and the plasma was separated. Erythrocytes were washed three times with phosphate buffered saline (PBS, pH 7.4).

### 2.4. Haemolysate Preparation

Erythrocytes were haemolysed with water in the following relation (1 : 1.5), then vortexed for 10 min, and centrifuged at 4000 rpm. Haemolysate was centrifuged at 16000 ×g for separation of erythrocyte membranes. Haemoglobin concentration was measured using Drabkin's method [17]. The crude haemoglobin without purification was used for estimation of conformational changes of this protein.

### 2.5. Spin Labelling of Crude Haemoglobin

**Figure 1:**
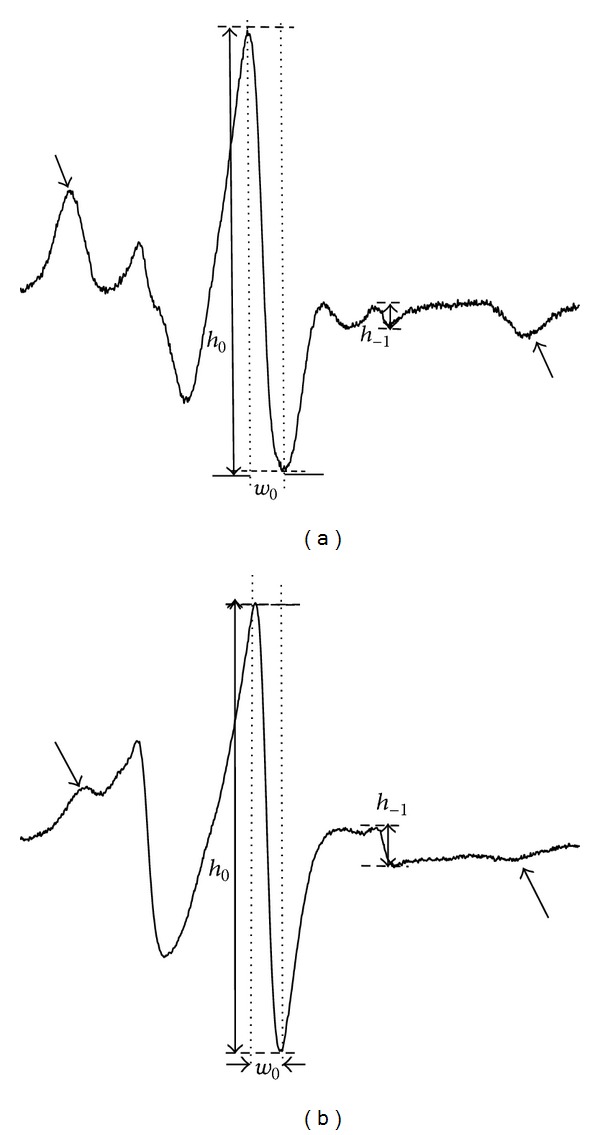
Electron paramagnetic resonance spectrum of (a) 4-maleimido-2,2,6,6,-tetramethylpiperidine-1-oxyl (MSL) and (b) 4-iodoacetamide-2,2,6,6,-tetramethylpiperidine-1-oxyl (ISL) attached to haemoglobin.

**Figure 2:**
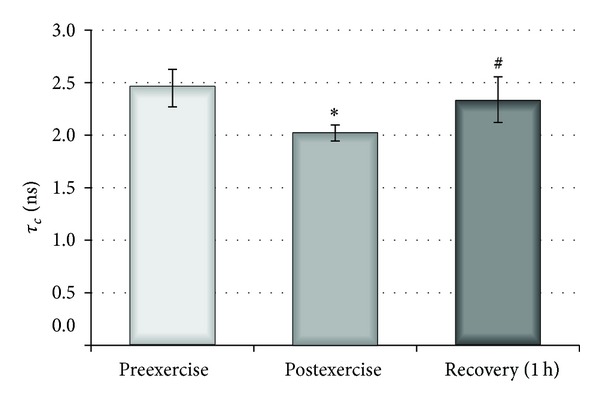
Influence of physical exercise on the mobility of internal erythrocyte peptides and proteins measured with MSL spin label before, after, and 1 hour after exercise. ∗ indicates significant difference in *τ*_*c*_ between preexercise and postexercise (*P* < 0.001); # indicates significant difference in *τ*_*c*_ between postexercise and 1 hour recovery (*P* < 0.01).

In order to investigate conformational changes of haemoglobin, MSL and ISL were used, which bind covalently to the -SH groups. The EPR spectra of spin labelled crude haemoglobin are shown in Figures 1 and 2.

Haemolysate was labelled using 0.1 mol/L ethanol solutions of MSL or ISL (50 : 1) and incubated for one hour at room temperature. The unbound spin labels were removed through dialysis using 20 mmol/L phosphate buffer, pH = 7.4, for 24 h at 4°C until the EPR signal in dialysis buffer disappeared. The effect of oxidative stress on the conformational changes of haemoglobin was studied by an estimation of the relative rotational correlation time *τ*_*c*_ for both spin labels.

The spectra of MSL and ISL attached to the crude haemoglobin indicated that these spin labels were immobilized. Estimation of the level of the mobility for these spin labels was possible by calculating relative rotational correlation times (*τ*_*c*_) according to the Kivelson formula (3) [18]:

(3) $$\begin{array}{c} \tau_{c}=kw_{0}\sqrt{\frac{h_{0}}{h_{-1}}}-1, \end{array}$$

where *τ*_*c*_ is time when the spin label undergoes full rotation, *k* is constant equal to 6.5 × 10^−10^ s, *w*_0_ is width of the midline of spectrum, *h*_0_ is height of the midline of spectrum, and *h*_−1_ is height of the high-field line of spectrum.

### 2.6. Spin Labelling of Erythrocyte Internal Components in Whole Erythrocytes

Changes of internal cytoplasmic peptides and proteins were investigated using a maleimide spin label in order to estimate the erythrocyte components mobility. More than 90% of the bound spin label remained in the cytosol and it can be inferred that MSL binds inside of the erythrocyte mainly with glutathione, less with haemoglobin and other proteins [19]. Erythrocytes were labelled with MSL during a one-hour period at room temperature. Next, any excess of spin label was removed by several washings with huge amounts of cold PBS, pH = 7.4, up to a diminishing of the EPR signal in the supernatant. From the EPR spectra the relative rotational correlation time was calculated according to formula (3).

### 2.7. Erythrocyte Internal Viscosity

Erythrocyte internal viscosity was monitored as described by Morse using Tempamine, which easily permeates the membrane and remains unbound inside the erythrocyte [20]. The relative rotational correlation times (*τ*_*c*_) was calculated using the Kivelson equation (3).

The erythrocyte internal viscosity were calculated according to the following formula:

(4) $$\begin{array}{c} \eta =\frac{{\tau_{c}}_{\left(\text{RBC}\right)}}{{\tau_{c}}_{\left({\text{H}}_{2}\text{O}\right)}}\eta_{{\text{H}}_{2}\text{O}}, \end{array}$$

where *τ*_*c*(RBC)_ is rotational correlation time for Tempamine inside the erythrocyte, *τ*_*c*(H_2_O)_ is rotational correlation time for Tempamine in water, and *η*_H_2_O_ is water viscosity equal to 1 cP.

### 2.8. EPR Measurements

EPR spectra were performed on the Bruker ESP 300 E spectrometer at room temperature (22 ± 2°C), operating at a microwave frequency of 9.73 GHz. The instrumental settings were as follows: the microwave power was 10 mW and the centre field was set at 3480 G, with a range of 80 G, a 100 kHz modulation frequency, a modulation amplitude of 1.01 G, and a time scan of 256 s.

### 2.9. Ascorbic Acid Measurements

The method is based on the ability of ascorbic acid reduction of the dye 2,6-dichlorophenolindophenol (indophenol) to a colourless compound [21]. The 10% m-phosphoric acid was added to 1 mL of haemolysate on ice bath to remove haemoglobin and other proteins. The mixture was centrifuged at 4000 ×g for 5 min. The citrate-acetate buffer (pH 4.15) with p-chloromercuribenzoic acid (p-CMB) sodium salt and indophenol solution at a concentration of 0.1 g/L were added to the supernatant. The absorbance of solution was measured after 30 s at 520 nm (*A*_1_) against water. Next, the ascorbic acid (in substantia) was added to the colourless solution (*A*_2_). The blank sample was prepared from a buffer containing p-CMB and indophenol, which was measured at 520 nm (*B*_1_) and repeated measurements were performed after the addition of ascorbic acid (in substantia) (*B*_2_):

(5) $$\begin{array}{c} \Delta A=\left(B_{1}-B_{2}\right)-\left(A_{1}-A_{2}\right). \end{array}$$

The concentration of ascorbate in the sample was estimated from the standard curve prepared for different concentrations of the ascorbic acid (in the range of 0–100 *μ*mol/L) and calculated as nmol/mg Hb.

### 2.10. Erythrocyte Membrane Preparation

Erythrocyte ghosts were prepared using the method described in [22]. Erythrocyte ghosts were used for the measurements of thiol groups, hydroperoxides, and acetylcholinesterase (AChE) activity. Membrane protein concentration was evaluated by the spectrophotometric method according to Lowry et al., using the Folin reagent [23].

### 2.11. Thiols Measurements

Erythrocyte membrane and plasma thiol groups were measured using the method of Ellman [24]. Samples were diluted with a 10 mmol/L phosphate buffer, pH 8.0, containing SDS. Following this, DTNB (5,5′-dithiobis(2-nitrobenzoic acid)) from a 10 mmol/L stock solution was added and samples were incubated for one hour at 37°C. The thiols reacted with DTNB to form anions with a strong yellow colour which were optically active at 412 nm. The basal optical activity of the samples was measured before the addition of DTNB. A calibration curve was prepared using different concentrations of reduced glutathione. The concentration of the thiol groups was calculated and expressed as *μ*mol/mg proteins of plasma or as nmol/mg proteins of erythrocyte membranes.

### 2.12. GSH Measurements

Reduced glutathione was analyzed in haemolysate using the method of Ellman [24]. The samples were treated with trichloroacetic acid (TCA) and the protein precipitate was removed by centrifugation. This method is analogous to determination of the protein thiol concentration. The concentration of GSH was estimated in mmol/packed cells (PC).

### 2.13. Peroxide Measurements

For the determination of the erythrocyte membrane and plasma hydroperoxides, the method with xylenol orange was employed. The reaction was based on the rapid oxidation of Fe(II) to Fe(III) in the presence of peroxides [25, 26]. 25 mmol/L ammonium iron(II) sulfate in 2.5 mol/L H_2_SO_4_ and 125 *μ*mol/L xylenol orange with 100 mmol/L sorbitol were mixed (1 : 100) to prepare a working solution. The samples were then mixed with a working reagent and incubated for 30 min in the dark. Reaction of Fe(III) with xylenol orange yielded a violet-coloured complex, which was quantified spectrophotometrically at 560 nm. The concentration of peroxides was expressed as *μ*mol/mg protein for erythrocyte membranes and as *μ*mol/L in the case of plasma.

### 2.14. Acetylcholinesterase Activity

The membrane acetylcholinesterase (AChE) activity was measured using Ellman spectrophotometric method [27].

### 2.15. The Reducing Potential of Plasma (RPP)

The reducing potential of plasma (RPP) was measured using a spectrophotometric method with 1,1-diphenyl-2-picrylhydrazyl (DPPH), a stable colour radical that reacts with antioxidants. Plasma was added to 150 *μ*mol/L DPPH in methanol (1 : 20) and incubated at room temperature for 30 min. The absorbance of samples was measured at 517 nm. Suppression of colour intensity was proportional to antioxidant content in plasma. A calibration curve was prepared from different Trolox concentrations in the range of 0–1000 *μ*mol/L [28]. The reducing potential of plasma was expressed as Trolox equivalents (in *μ*mol/L of plasma).

### 2.16. Plasma Viscosity

Plasma viscosity was monitored as described by Morse using Tempamine [20]. Plasma viscosity was calculated using data obtained from EPR spectra according to formula (4).

### 2.17. Plasma Lipid Peroxidation

Lipid peroxidation was measured using the method with thiobarbituric acid (TBA) described in [29].

### 2.18. Plasma Carbonyl Compounds

Plasma protein carbonyl content was estimated using 2,4-dinitrophenylhydrazine (DNPH) [30].

### 2.19. Statistical Analysis

All measurements are presented as means [±] and standard deviation (SD). For statistical analysis, we used the SPSS package. A repeated-measures analysis of variance (ANOVA) with a Tukey test was used to evaluate the differences between the means. Statistical significance was accepted at *P* < 0.05.

## 3. Results

The investigated parameters associated with oxidative stress were monitored in whole red blood cells, isolated haemoglobin, membranes, and plasma. This was done for blood collected at three time points: before exercise, immediately after exercise, and one hour after exercise.

The amount of red blood cells and haemoglobin concentration were determined as parameters that indirectly indicated oxygen transport by blood. Lactate concentration was measured as a muscle metabolite. Another important parameter was that the level of plasma volume changes after exercise, and this was determined by the original method proposed by Dill and Costill [16].  Table 1 shows the physicochemical parameters of blood at different stages: before, immediately after, and one hour after exercise. Significant alterations in all the studied parameters were observed immediately after exercise. One hour later, all returned to the original control values except in the case of the plasma volume.

EPR spectroscopy was used to investigate conformational changes of haemoglobin, internal viscosity of erythrocytes, and plasma viscosity. Tempamine was used to investigate changes in internal viscosity of erythrocytes. Measurements of intercellular viscosity were based on calculation of relative rotational correlation time for this spin label [20]. Our results did not show statistically significant changes at different time points after exercise. Moreover, the results obtained for Tempamine were confirmed by detection of mobility of erythrocyte internal components, for example, proteins, and peptides using maleimide spin label. Analysis of the maleimide spectra and calculation of the relative rotational correlation times showed nonsignificant changes in this parameter immediately after exercise and one hour later in comparison to control.

Conformational changes of haemoglobin were estimated in cell lysates using iodoacetamide and maleimide spin labels, both of which covalently bind to -SH groups on cysteine-93 of *β*-globin chains [31, 32].  Figure 1 shows the EPR spectra of haemoglobin labelled with MSL and ISL [33]. Both MSL- and ISL-labelled haemoglobin spectra had two different spatial orientations relative to the haemoglobin molecule [31, 32]. However, both spin labels attached to the haemoglobin exhibited considerable differences in local anisotropic motion with respect to the site to which they were attached. The broad signals marked by the arrows are due to maleimide being strongly immobilized at -SH groups on cysteine 93 (Figure 1). A relative rotational correlation time was calculated from the spectra for both labels. This parameter was significantly decreased for MSL immediately after exercise in comparison to controls (*P* < 0,001), but one hour later an increase in *τ*_*c*_ was found (Figure 2). In the case of ISL, there were no significant changes at any of the investigated time points. These results suggest that the acute exercise caused reversible changes in the conformational state of globin chain of haemoglobin.

**Figure 3:**
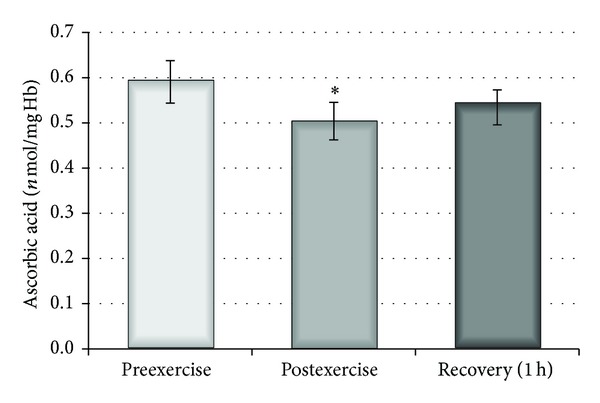
Changes in ascorbic acid concentration inside the erythrocytes before, after, and 1 hour after exercise. ∗ indicates significant difference between preexercise and postexercise (*P* < 0.005).

Investigation of low molecular weight of antioxidants inside the erythrocytes, for example, glutathione and ascorbic acid, showed a significant decline of ascorbic acid immediately after exercise (*P* < 0,005) and slight increase one hour later (Figure 3). The level of glutathione increased just after exercise and decreased one hour after exercise, but these data were statistically insignificant.

**Figure 4:**
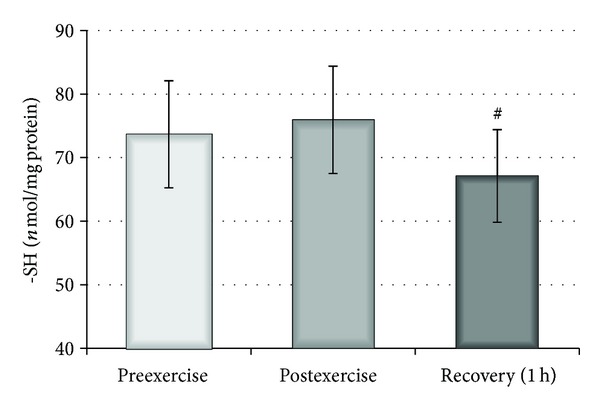
Influence of physical exercise on thiol group concentration of erythrocyte membranes measured before, after, and 1 hour after exercise. # indicates significant difference between postexercise and 1 h recovery (*P* < 0.05).

The levels of thiol groups, peroxides, and acetylcholinesterase activity in the erythrocyte membrane were also estimated. The concentration of -SH groups was significantly lower (*P* < 0.05) one hour after exercise in comparison to the controls before and immediately after exercise (Figure 4). The level of erythrocyte membrane peroxides was not changed significantly at any of the investigated time points after exercise. The erythrocyte acetylcholinesterase activity is a useful marker of oxidative stress. This enzyme is located on the outer surface of the plasma membrane and its activity is generally decreased by oxidizing agents. However, AChE activity in membranes was only insignificantly lower immediately upon exercise and one hour after exercise.

**Figure 5:**
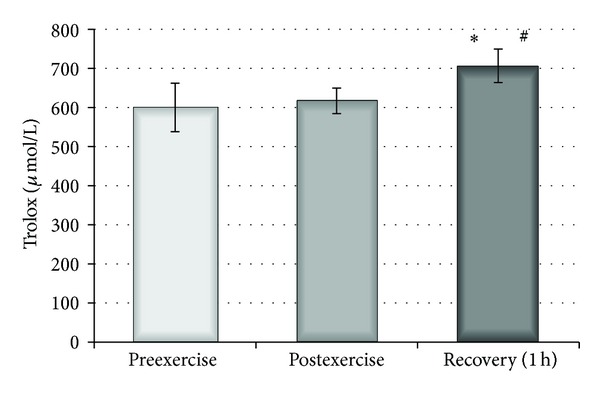
Effect of physical exercise on reducing potential of plasma estimated before, after, and 1 hour after physical exercise. ∗ indicates significant difference between preexercise and 1 hour recovery (*P* < 0.0002); # indicates significant difference between postexercise and 1 h recovery (*P* < 0.001).

**Figure 6:**
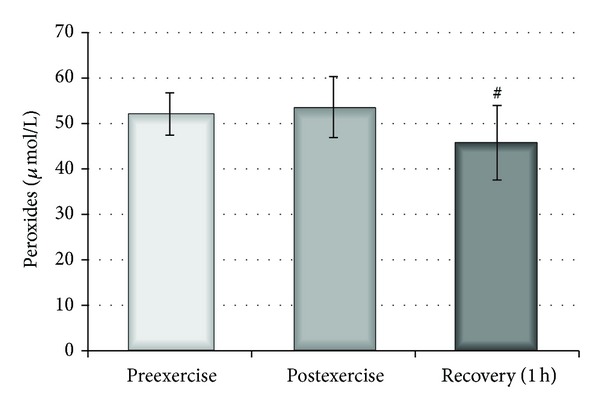
Effect of physical exercise on peroxide concentration in plasma measured before, after, and 1 hour after physical exercise. # indicates significant difference between postexercise and 1 h recovery (*P* < 0.02).

**Figure 7:**
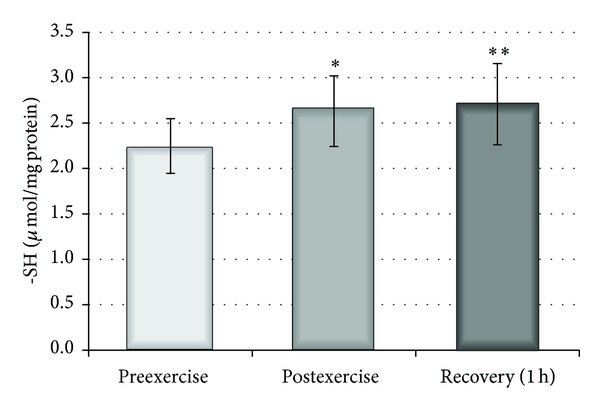
Effect of physical exercise on thiol group concentration in plasma measured before, after, and 1 hour after physical exercise. ∗ indicates significant difference between preexercise and postexercise (*P* < 0.02); ∗∗ preexercise and 1 hour recovery (*P* < 0.01).

The effect of a single bout of exercise on the plasma parameters was also examined. An increase in the reducing potential of plasma, measured by DPPH radical using the spectrophotometric method, was observed 1 hour after exercise (*P* < 0.05) and, additionally, significant differences in RPP levels were detected between the time points both immediately after and one hour after exercise (*P* < 0.05) (Figure 5). Nevertheless, analysis of the parameters of plasma related to oxidative stress revealed a non-significant increase in TBARS level. This change was accompanied by a significant decrease (*P* < 0.02) in plasma peroxides observed both immediately after and 1 hour after exercise (Figure 6). Furthermore, neither the levels of carbonyl compounds nor the viscosity of plasma changed following a single bout of exercise (data not shown). The total level of plasma thiol groups was significantly elevated immediately after exercise (*P* < 0.002) and one hour after exercise (*P* < 0.01) compared with preexercise values (Figure 7).

## 4. Discussion

The question of the role of ROS and their interactions with cell components and physiopathological consequences following physical exhaustion is an important issue in free radical biochemistry. Generally, the intensity of ROS generation during exercise depends on the level of antioxidant enzyme activities, diet, time and intensity of exercise, and oxygen consumption.

Commonly, investigations of alterations in plasma, muscles, and other tissues in body organs are of relevance to athletes. It seems that a study on a young untrained man may be useful in explaining changes in the blood of nonathletes undergoing rehabilitation.

In our study, young, untrained, healthy males were subjected to physical exhaustion by a single bout of exercise. We described early mechanisms of oxidative stress development in erythrocyte membranes reflected by changes in their properties immediately after exercise and during recovery time [12].

Lactate is a metabolic product of glucose conversion in muscles during glycolysis and its levels have been studied in sport medicine since the 1930s. It is a useful parameter for short exercise experiments. An approximate fourfold increase in this parameter was observed immediately after exercise, and a decrease in the control value was detected after one hour of recovery time. In addition, a significant increase in number of erythrocytes, haematocrit, and concentration of haemoglobin was observed immediately after exercise. One hour after exercise, the levels of these parameters were comparable to the control values. These observations are in agreement with other studies [34].

Another important parameter was the change in plasma volume. This parameter reflected changes in the total serum protein concentration and indicated water leaching from the vascular system during exercise. More pronounced changes in ΔPV were observed immediately after exercise, while one hour later the changes were moderate.

The changes in internal components of erythrocytes, for example, in cytoplasmic peptides and proteins including haemoglobin, can occur as a consequence of acute exercise. Because haemoglobin remained in continuous interaction with increased oxygen supply, the changes in the conformational state of this molecule were studied.

Erythrocytes can be damaged by both internal and external ROS sources. Internally, haemoglobin may be autooxidized to methaemoglobin resulting in the production of superoxide anions, which are precursors of other reactive oxygen species (hydrogen peroxide, hydroxyl radical, and singlet oxygen) [35]. Externally, ROS (including hypochlorous acid) can be released by activated neutrophils. Strong oxidative agents can easily oxidize cellular proteins and unsaturated lipids. However, erythrocytes can be protected against ROS by enhanced activity of antioxidant enzymes and high concentration of glutathione and other low molecular weight antioxidants. It seems that a higher consumption of oxygen by the body during exercise can increase the level of the superoxide anion released from haemoglobin inside red blood cells as well as increase the release of ROS by activated neutrophils in plasma. Under these conditions RBC may be damaged by internal and external sources of ROS. Thus, haemoglobin can also be considered as a potent free radical catalyst. This fact motivated us to investigate the internal components of RBC.

Potential changes in the dynamics of red blood cell components after exercise were studied by electron paramagnetic resonance spin labelling spectroscopy. Spin labels were used in the investigation of internal viscosity, the mobility of internal components, and the conformational state of haemoglobin. They bound to macromolecules and played the role of reporting groups providing information about the changes in the microenvironment of the nearest region.

Two spin labels (Tempamine, MSL) were applied for the analysis of internal viscosity and the physical state of RBC internal components. With the usage of Tempamine a tendency to increase the internal viscosity of erythrocytes both immediately after exercise and one hour later was observed, but these results were not statistically significant. It was shown that at physiological pH maleimide spin label reacts with thiol groups of peptides and proteins [36]. MSL easily penetrates cellular membrane of erythrocytes and attaches mainly to glutathione [19]. A slight increase in the motion of this label was found immediately after exercise, but again in this case the results were not statistically significant. Generally, any modulation following a single bout of exercise in young, untrained males did not influence the properties of the internal fluid of erythrocytes.

Possible conformational changes of haemoglobin can be estimated using maleimide and iodoacetamide spin labels. A decrease in relative rotational correlation time value was detected for MSL-labelled haemoglobin, which indicates conformational changes in the structure of globin chains immediately after exercise. However, one hour after exercise the observed tendency returned to normal levels. This result may suggest reversible conformational changes in the haemoglobin chain. However, the modulation of conformational properties of Hb following a single bout of exercise in young, untrained males did not influence internal fluid properties.

Statistically significant changes in the concentration of ascorbic acid were detected inside erythrocytes, shown by a decrease immediately after exercise and an increase one hour after exercise. It explains the fact that following a single bout of exercise the internal defence system of the cells was activated as a result of elevated oxidative stress in the plasma. Tissue ascorbate concentration exceeds its plasma levels by three to ten times. It is possible that during oxidative stress (strenuous exercise) ascorbate might be released from tissues to plasma and subsequently to erythrocytes because equilibrium between its concentration outside and inside RBCs exists.

Decreased level of GSH inside erythrocytes was associated with a significant decrease of -SH groups in the membrane proteins one hour after exercise as a consequence of induced oxidative stress.

Erythrocyte acetylcholinesterase is a useful marker of oxidative stress. The inhibition of this enzyme by oxidizing agents, such as hydrogen peroxide and lipid peroxides, was described in the 1960s [37]. A decline in AChE activity was also observed in ischemic patients and in the process of ageing [38, 39]. Moreover, we found that the acetylcholinesterase activity in the cellular membrane exhibited a decreasing tendency, which also indicates the presence of oxidative stress in the cellular membrane. On the other hand, peroxide levels in the membrane did not change at any of the investigated time points.

In our study, we also observed an insignificant increase of TBARS in the plasma. This result is in line with numerous earlier studies on both maximal and submaximal exercise [2, 40, 41]. Furthermore, similar to previous studies, hydroperoxide levels were found to be significantly decreased [2, 40, 41]. It is possible that part of the hydroperoxides, through a process of fragmentation, is converted to aldehydes. Nevertheless, an approximate twofold increase in MDA plasma levels after exercise was observed by Sureda et al. [42]. In addition, some other studies found elevated or unchanged hydroperoxide levels [43, 44].

Moreover, the level of carbonyl compounds or plasma fluidity did not significantly change at any of the investigated time points after exercise. However, while an increase in protein carbonyls was observed by some authors, others did not report such results in trained subjects [45, 46].

Generally, a decrease in antioxidant capacity was observed in response to strenuous exercise [47, 48]. In our study, there was no change in the antioxidant capacity of plasma immediately after exercise. Similar results were reported by Waring et al. [49]. However, a statistically significant increase in plasma antioxidant capacity was detected one hour after exercise. This observation confirms results from earlier findings. There are a number of studies where an increase in plasma antioxidant capacity was found during recovery time by applying different biochemical methods [12, 40, 49].

We also observed an increase in total thiols both immediately after and one hour after exercise. Elevation of blood plasma thiols was also reported by Rajguru et al., who suggested that an increase in thiols can be related to triggering the repair system in the damaged RBC membrane [50]. Therefore, an increase of both antioxidant capacity and total thiols suggests the existence of strong defence mechanisms in plasma.

We hypothesize that the oxidative stress generated during a single bout of exercise is mainly caused by shear stress, which leads to neutrophil activation and ROS release. The level of myeloperoxidase in blood was reported to double after exercise [42, 51]. This result shows that hypochlorous acid is one of the oxidizing agents in plasma. Shear stress may lead to ROS generation and the activation of cyclooxygenase 2 and endothelial cell nitric oxide synthase [52, 53].

Exercise increases heart rate and blood flow, which together lead to vascular shear stress and ROS generation by an endothelium-dependent mechanism [52]. The expression of endothelial nitric oxide synthase (eNOS) and nitric oxide production as a consequence of shear stress have also been extensively discussed [53, 54]. Thus, peroxynitrite can be another molecule responsible for oxidative stress in plasma.

The increase in reducing plasma potential and in thiols concentration in response to oxidative stress provides evidence for mobilization of a defence system and can be explained as an adaptation reaction of the human body and the vascular system to exercise. We conclude that a single bout of exercise mobilizes antioxidant defence systems in plasma and erythrocytes.

In summary, RBCs provide an effective protection system against externally triggered oxidative stress. However, it seems that, during maximal or prolonged submaximal exercise, RBCs may be exposed to greater damage from externally induced oxidative stress.

Such studies are arguably useful in explaining changes in the blood of nonathletes undergoing rehabilitation.

## Figures and Tables

**Table 1:** Effect of physical exercise on lactate, haemoglobin concentration as well as haematocrit, and delta plasma volume (ΔPV) before, after, and 1 hour after exercise.

Parameter	Preexercise $\overset{-}{x}\;$ ± S.D	Postexercise $\overset{-}{x}\;$ ± S.D	Recovery (1 h) $\overset{-}{x}\;$ ± S.D
Lactate (mmol/L)	2.47 ± 0.78	11.19 ± 1.78*	3.33 ± 0.80^#^
Haemoglobin (g/dL)	15.44 ± 0.87	16.75 ± 0.78**	15.24 ± 0.87^#^
Haematocrit (%)	45.56 ± 2.22	49.72 ± 2.40**	44.90 ± 2.46^#^
ΔPV (%)		−14.47 ± 3.95	3.02 ± 4.19

*Significant difference between preexercise and postexercise (*P* < 0.0002); **preexercise and postexercise (*P* < 0.0005); ^#^significant difference between post-exercise and 1 h recovery (*P* < 0.0002).
